# AI-Enabled Inclusive Oral-Systemic Integrated Care (AI-IOC+): An Equitable, Learning Framework for Managing Polypharmacy, Multimorbidity, and Special Health Needs Throughout Life

**DOI:** 10.7759/cureus.107497

**Published:** 2026-04-21

**Authors:** Sumeet Agarwal, Laresh N Mistry, Ashwin M Jawdekar, Deepak Sharma, Harsh Mishra, Sayem A Mulla

**Affiliations:** 1 Prosthodontics, Bharati Vidyapeeth Dental College and Hospital, Navi Mumbai, IND; 2 Pedodontics and Preventive Dentistry, Bharati Vidyapeeth Dental College and Hospital, Navi Mumbai, IND; 3 Conservative Dentistry and Endodontics, Bharati Vidyapeeth Dental College and Hospital, Navi Mumbai, IND; 4 Orthodontics and Dentofacial Orthopedics, Bharati Vidyapeeth Dental College and Hospital, Navi Mumbai, IND; 5 Dentistry, Independent Researcher, Kalyan, IND

**Keywords:** ai-ioc+, artificial intelligence, dentistry, health, health technology

## Abstract

Increased polypharmacy, ineffective resource use, and worse results for people with chronic illnesses, especially older folks and those with specific healthcare needs, are all consequences of the fragmentation of dental and medical services. Few frameworks integrate AI with oral-systemic integration and a life-course, equity-centered approach, despite the fact that AI offers scalable potential to integrate diverse health data, improve clinical decision-making, and promote proactive population health management. Predictive risk modeling for oral-systemic disease clustering, adaptive patient and caregiver interfaces for special populations, AI-assisted clinical decision support, including medication reconciliation and contraindication alerts, and a learning health-system feedback mechanism to optimize care pathways are all included in the proposed AI-Enabled Inclusive Oral-Systemic Integrated Care (AI-IOC+) framework. The AI-IOC+ model, which places a strong emphasis on sustainability, fairness-by-design, data interoperability, and governance, seeks to improve early detection and prevention of systemic complications associated with oral disease, improve access and continuity of care for vulnerable populations, lower adverse drug events through AI-supported deprescribing, and increase healthcare efficiency by reducing duplication. With future prospects including focused scoping reviews, stakeholder co-design pilots, and health economic assessments to enable widespread implementation, this approach offers policymakers, academics, and healthcare systems a useful road map.

## Introduction and background

Health systems and economies throughout the world are under unprecedented pressure due to the rising prevalence of chronic noncommunicable diseases (NCDs), such as diabetes, respiratory conditions, oral problems, and cardiovascular diseases [1]. More than one in three people worldwide suffer from multimorbidity, or the coexistence of numerous chronic illnesses, and its incidence is predicted to increase with population aging and changes in lifestyle. Widespread polypharmacy, which is the concurrent use of several drugs and raises the risk of adverse drug events, hospitalizations, and treatment nonadherence, coincides with this rise [2]. Health systems continue to tackle these issues by dividing the dental, medical, and social care domains into disjointed, disease-specific silos. In addition to maintaining redundancy and inefficiencies, this division ignores the oral-systemic links that are now understood to be vital to general health [3].

Systemic problems such as diabetes, cardiovascular disease, chronic obstructive pulmonary disease, and neurodegenerative disorders have many biological and behavioral risk factors with oral diseases. For example, edentulism is connected to frailty and malnutrition in older persons, but periodontitis has been linked to poor glycaemic management and increased cardiovascular risk [4]. Even in nations with sophisticated primary healthcare systems, oral healthcare delivery is still mainly left out of mainstream integrated care (IC) frameworks. A strategy to enhance patient outcomes, reduce system fragmentation, and encourage economical chronic illness treatment is to integrate oral and systemic health into a unified care paradigm [5].

The World Health Organization and other international frameworks promote integrated care, which aims to provide coordinated, person-centered services throughout the prevention, treatment, and long-term support continuum. However, due to organizational silos, mismatched data systems, and a lack of interprofessional collaboration, implementation is still inconsistent despite conceptual maturity [6]. Furthermore, a variety of populations that face disproportionately high hurdles to care continuity and accessibility, including children, adolescents, older adults, and those with impairments or complex health needs, are frequently ignored by traditional integration approaches [7].

New developments in digital health and artificial intelligence (AI) offer a revolutionary opportunity to operationalize integrated care on a large scale. AI's ability to enhance decision-making, predictive analytics, and data harmonization can help close information and coordination gaps between different service levels and disciplines [8]. Clinical decision support, risk stratification, and drug optimization are examples of early medical applications that show quantifiable increases in efficiency and safety. Despite the availability of extensive dental, radiographic, and clinical data that could improve the management of multimorbidity and polypharmacy, the integration of AI into oral-systemic frameworks remains largely unexplored [9].

We suggest the AI-Enabled Inclusive Oral-Systemic Integrated Care (AI-IOC+) framework, a learning, equitable paradigm that integrates lifetime inclusiveness, artificial intelligence, and oral-systemic integration, to close this crucial gap. The AI-IOC+ strategy presents AI as a digital integrator that optimizes pharmaceutical safety, enables real-time communication between dental and medical information, and constantly improves care through feedback learning. Crucially, it incorporates sustainability, fairness, and accessibility as cross-cutting principles, ensuring that vulnerable groups such as those with disabilities and those from low-income backgrounds benefit from digital transformation. AI-IOC+ provides a practical road map for high-performing, patient-centered, and resilient healthcare systems by connecting preventative, clinical, and social determinants of health. This coincides with worldwide moves toward precision public health and value-based integrated care.

## Review

Methodology

Using an organized yet adaptable methodology, this narrative review was carried out to compile the available data on AI-enabled oral-systemic integrated care. Systematic searches of electronic databases such as PubMed/MEDLINE, Scopus, Web of Science, and Google Scholar were conducted to find pertinent material. To ensure thorough coverage of the subject, additional sources were obtained by manually checking the reference lists of important papers. Peer-reviewed publications, systematic and narrative reviews, clinical trials, and pertinent conceptual or policy papers on artificial intelligence in healthcare, oral-systemic health integration, multimorbidity, polypharmacy, and digital health frameworks were among the inclusion criteria. Studies that addressed a variety of populations across the lifespan and were published in English were taken into consideration. Non-peer-reviewed sources, conference abstracts without full texts, research unrelated to AI or integrated care frameworks, and papers concentrating only on discrete dental or medical outcomes without systemic linkage were among the exclusion criteria. To offer a thorough and interdisciplinary picture, a variety of study types were included, including systematic reviews, meta-analyses, observational studies, clinical trials, and excellent narrative reviews. The final review contained a total of 38 studies after screening and eligibility evaluation. The suggested AI-IOC+ framework was developed using a qualitative analysis and theme synthesis of the selected literature.

Conceptual framework: AI-enabled inclusive oral-systemic integrated care (AI-IOC+) model and structural layers of integration

The AI-IOC+ model is envisioned as an adaptable, digitally enhanced framework that integrates lifetime inclusion, oral-systemic integration, and artificial intelligence into a unified, people-centered care environment [10]. The strategic use of AI for dynamic decision support, interoperability, and population health intelligence expands the model, which is based on the concepts of integrated care coordination, continuity, comprehensiveness, and person-centeredness [11]. This framework operates on four interdependent layers, which ensure a continuum from prevention to precision intervention

Preventive and Population Layer

Fundamentally, the methodology integrates oral, medical, behavioral, and social determinants of health with AI-driven predictive analytics to prioritize early risk detection. Large-scale dataset-trained machine-learning algorithms can predict oral-systemic illness trajectories, assist in targeted preventive treatments, and stratify individuals and subgroups by multimorbidity risk [12]. For example, AI models have shown the ability to predict cardiovascular risk using periodontal and dental radiograph data, demonstrating the potential of oral datasets for systemic prediction [13].

Clinical and Diagnostic Layer

Bidirectional integration between dental and medical treatment is made possible by this layer. To provide a thorough health snapshot, artificial intelligence (AI) tools such as clinical decision support systems (CDSS) and natural language processing (NLP) algorithms harmonize patient data from pharmacy databases, dental management systems, and electronic health records (EHRs). Through automated alarms and AI-assisted medication reconciliation, the integration of pharmacogenomic and medication adherence data promotes safer prescribing and lowers risks associated with polypharmacy [14]. When combined with systemic data, AI-assisted radiomics and deep learning in dentistry can detect comorbidity patterns that would otherwise go unnoticed in siloed systems. These capabilities currently improve diagnosis accuracy for caries, bone loss, and oral cancers [15].

Supportive and Rehabilitative Layer

The AI-IOC+ approach acknowledges that biomedical therapy is not the only way to manage chronic illnesses. Here, chatbots and virtual assistants with AI capabilities offer behavioral reinforcement, medication reminders, and dental hygiene coaching, all of which are very helpful for older citizens and those with impairments [16]. By customizing treatments to cognitive and physical capacities, these technologies reduce disparities in self-management support and promote autonomy, continuity, and adherence [17].

System-Level and Governance Layer

AI-IOC+ incorporates workforce, governance, interoperability, and ethical data frameworks as system enablers at the macro level. It encourages the adoption of federated learning models, which let organizations train AI systems together without jeopardizing patient privacy. This decentralized learning promotes shared value, openness, and trust across institutions [18]. Additionally, this layer's policy procedures provide ethical AI governance, regulatory scrutiny, and fair resource distribution across various demographic groups. The concept emphasizes digital transformation as a driver of universal health coverage, which is in line with the WHO's worldwide plan on digital health 2020-2025 [19].

Inclusive design across the lifespan and diverse populations

Inclusion is necessary for true integration. The AI-IOC+ paradigm incorporates an equity-by-design philosophy that acknowledges intercultural diversity, age, and aptitude as factors influencing oral and overall health outcomes [20]. AI-assisted behavioral monitoring can help identify comorbid behavioral problems that affect nutrition and dental hygiene, identify developmental abnormalities, and predict caries risk in pediatric and adolescent populations [21]. In senior populations, AI-enhanced monitoring of masticatory performance, medication usage, and nutritional status offers early diagnosis of frailty and aspiration risk, permitting integrated geriatric-dental treatment. AI-supported caregiver communication, speech-driven records, and adaptable interfaces enhance accessibility and lower dependence barriers for people with impairments [22].

Interoperability and data integration

The development of interoperable data ecosystems is essential to AI-IOC+. These use common ontologies like Systematized Nomenclature of Medicine-Clinical Terms (SNOMED-CT) and FHIR (Fast Healthcare Interoperability Resources) to integrate datasets related to dentistry, medicine, pharmacy, and social care [23]. Continuous learning is made possible by this unified data architecture, which enables feedback loops to improve predicted accuracy and quality enhancement. In order to manage multimorbidity and maximize population-level treatments, it turns scattered encounters into longitudinal health narratives [24].

Implementation pathway and evaluation metrics

A staged implementation plan is necessary for operationalizing AI-IOC+. Assessing labor competence, data infrastructure, and digital maturity is known as a readiness assessment. Installing AI-driven, interoperable modules in a few health networks is known as pilot integration. Scale-up and learning loops for incremental improvement based on real-world data, guided by assessment metrics like polypharmacy reduction, cost savings, and patient outcomes [25]. A triple goal framework that improves patient experience, population outcomes, and provider satisfaction, and optimizes cost efficiency, is used for evaluation. Adaptive recalibration, which embodies the "learning health system" philosophy, is made possible by ongoing feedback from patients, professionals, and systems [26].

Comparative advantages over existing IC models

By incorporating artificial intelligence, oral-systemic connections, and inclusive, lifespan-focused care, the AI-IOC+ framework extends conventional models and is a strategic progression of integrated care [27]. AI-IOC+ provides a dynamic, learning, and equitable system that can address multimorbidity, polypharmacy, and healthcare access disparities across diverse populations, in contrast to traditional integrated care (IC) approaches that often operate in silos or narrowly focus on specific chronic diseases [28].

Enhanced data integration and decision support

Conventional IC models have limited scalability and accuracy since they primarily rely on human interpretation of patient data and manual coordination. AI-IOC+ provides real-time clinical decision assistance by harmonizing medical, dental, pharmacy, and social care records using machine learning and natural language processing [29]. This feature is especially helpful for managing polypharmacies, as AI-assisted warnings and deprescribing suggestions can maximize therapeutic efficacy, enhance adherence, and avoid adverse medication events [30].

Predictive and preventive capacity

AI-IOC+ enables early diagnosis of comorbid illnesses, including diabetes, cardiovascular disease, and cognitive decline, by incorporating oral health data into systemic risk algorithms. A fundamental tenet of precision public health, this predictive power enables focused preventative interventions, shifting health systems from reactive to proactive treatment [31]. There is a significant gap in the thorough risk categorization, since current IC models seldom ever use dental data [32].

Lifespan inclusivity and equity

AI-IOC+ specifically includes children, adolescents, older adults, and people with disabilities, in contrast to traditional IC frameworks that mostly concentrate on adult populations or single care settings [33]. In line with the socioeconomic determinants of health and universal health coverage principles, adaptive AI interfaces, caregiver integration tools, and behaviorally customized treatments lessen disparities in access and adherence [34].

Implementation challenges and limitations

AI-IOC+ has potential; however, there are a number of implementation issues. Standardized coding methods like FHIR and SNOMED-CT are necessary since data interoperability is still a significant obstacle. Fairness-by-design frameworks must thoroughly handle ethical issues, such as algorithmic bias, informed consent, and privacy [35]. Adoption also depends on workforce preparedness and digital literacy; physicians need to be taught to understand AI-generated insights and incorporate them into patient-centered decision-making. Scalability may be limited by infrastructure and cost limitations, especially in low-resource environments, highlighting the need for progressive, pilot-tested deployment tactics [36].

Policy and research implications

Clear avenues for policy innovation are provided by AI-IOC+. Federated AI models may be used by health systems to assist national digital health objectives by facilitating multi-institutional collaboration without sacrificing privacy. Evaluation metrics should combine the quadruple aim patient experience, population results, provider satisfaction, and system efficiency to provide a thorough assessment of efficacy [37]. Human-centered design studies to maximize AI usability across populations, ethical evaluations to protect equality and privacy in AI-mediated IC, and longitudinal studies to evaluate therapeutic and economic effects are among the research goals [38].

Future directions

Scalability and adaptability are included in the AI-IOC+ paradigm. Preventive and rehabilitative treatment can be further improved by integrating with emerging technologies, including wearables, home-monitoring sensors, and tele-dentistry platforms. Furthermore, continuous learning loops enable AI systems to be iteratively improved, guaranteeing responsiveness to changing system requirements and population health trends. In the end, AI-IOC+ represents a paradigm shift in integrated care that is egalitarian, patient-centered, data-driven, and ready to meet the demands of ever-more complex health systems.

## Conclusions

In order to enhance lifetime health outcomes, especially for vulnerable and marginalized groups, the AI-Enabled Inclusive Oral-Systemic Integrated treatment (AI-IOC+) framework is a forward-thinking concept that uses artificial intelligence to integrate medical and dental treatment. It improves multimorbidity treatment, lowers polypharmacy, supports proactive disease prevention, and fosters fairness and inclusivity by utilizing predictive analytics, clinical decision support, and adaptive patient interfaces. AI-IOC+ provides better data integration, learning-system flexibility, and moral, interoperable governance to lessen health inequities as compared to traditional integrated care models. Phased, evidence-based policies, workforce training, a strong digital infrastructure, and ongoing assessment utilizing the quadruple goal are all necessary for its effective implementation. All things considered, AI-IOC+ offers a sustainable, egalitarian, and scalable model for precision public health and integrated healthcare systems that are prepared for the future.
